# Influence of experience, tenure, and organisational preparedness on nurses' readiness in responding to disasters: An exploration during the COVID-19 pandemic

**DOI:** 10.7189/jogh.13.06034

**Published:** 2023-08-14

**Authors:** Mariusz Goniewicz, Amir Khorram-Manesh, Anna Włoszczak-Szubzda, Dorota Lasota, Ahmed M Al-Wathinani, Krzysztof Goniewicz

**Affiliations:** 1 Department of Emergency Medicine, Medical University of Lublin, Lublin, Poland; 2 Department of Surgery, Institute of Clinical Sciences, Sahlgrenska Academy, Gothenburg University, Gothenburg, Sweden; 3 Learning and leadership for Healthcare Professional, Institute of Health and Care Sciences, Sahlgrenska Academy, Gothenburg University, Gothenburg, Sweden; 4 Gothenburg Emergency Medicine Research Group (GEMREG), Sahlgrenska Academy, Gothenburg University, Gothenburg, Sweden; 5 Faculty of Human Sciences, University of Economics and Innovation, Lublin, Poland; 6 Department of Experimental and Clinical Pharmacology, Medical University of Warsaw, Warsaw, Poland; 7 Department of Emergency Medical Services, Prince Sultan bin Abdulaziz College for Emergency Medical Services, King Saud University, Riyadh, Saudi Arabi; 8 Department of Security, Polish Air Force University, Dęblin, Poland

## Abstract

**Background:**

The coronavirus 2019 (COVID-19) pandemic has placed unprecedented challenges on the nursing practice, particularly in Poland. Nurses, as crucial healthcare service providers, have faced organisational disruptions, altered working conditions, and heightened professional anxieties.

**Methods:**

We undertook a comprehensive survey across all medical centres in Lublin, Poland in 2020 to understand nurses' attitudes towards their roles and working conditions during the pandemic. This involved 470 nurses completing a questionnaire which focused on four pivotal areas: readiness to be on call in a disaster situation (even when not formally asked); willingness to work overtime in a disaster without additional compensation, preparedness to undertake health risks by caring for individuals with infectious diseases or exposure to hazardous substances, and willingness to be transferred to other departments during a disaster.

**Results:**

We found that excessive workload, fear of infection, and feelings of helplessness significantly influenced nurses' readiness to work overtime, particularly when unpaid. We also presented the ethical dilemmas that nurses encountered during the pandemic and how these dilemmas affected their decision-making processes. We further explored the impact of variables such as nurses' professional experience, tenure, and level of organisational preparedness on their readiness to respond to crisis situations.

**Conclusions:**

Gaining an understanding of nurses' perspectives is key for formulating strategies to bolster their professional engagements and resilience during crises. Addressing these issues can help build a more robust and well-prepared healthcare system that can effectively navigate future crises.

Nursing, a vital element of healthcare systems globally, is undergoing rapid changes. It is a discipline dedicated to providing individuals, families, and communities the care needed to maintain or achieve optimal health and quality of life [1]. Today, nurses are more than just healthcare providers; they are change agents, patient advocates, leaders, and researchers.

In Poland, the nursing profession has gained substantial recognition. Upon qualification, nurses are no longer viewed as ancillary but as integral partners in the therapeutic team. Their responsibilities have extended beyond traditional caregiving duties, encompassing the recognition of patient health needs, the planning and provision of nursing care, preventive, diagnostic, therapeutic, and rehabilitation services, the performance of medical emergency activities within a predefined scope, and health education and promotion [1]. These tasks represent a transformation in the nursing profession from a vocation to a profession, a shift facilitated by higher education levels and improved professional competencies.

This change has not come without increased responsibilities and ethical considerations. Nurses are now accountable for their decisions about patient care, driven by established medical knowledge, the Code of professional ethics, and the applicable legal standards. These ethical principles, derived from universal values, bind nurses to respect patient rights and uphold the dignity of the nursing profession, and are not mere guidelines but an essential minimum for the nursing profession, with patient responsibility being their foremost duty [2].

These changes have led to nurses adopting new roles, particularly in disasters and public health emergencies (DPHEs) [3], in during their pivotal role, combined with their clinical and emotional competence, makes them indispensable.

The coronavirus 2019 (COVID-19) pandemic has highlighted the complex and challenging nature of DPHEs, presenting unforeseen challenges to everyday nursing practice, particularly in public healthcare settings. Working directly with SARS-CoV-2 infected patients, nurses in public healthcare institutions grappled with multiple risks, including the fear of infection, the unpredictability of events, feelings of helplessness, and concerns about performing their professional duties [4]. In this study, we focus on the experiences of nurses in public healthcare settings to explore the challenges and dilemmas they faced during the COVID-19 pandemic.

Several recent studies have explored the challenges that nurses faced during the COVID-19 pandemic, such as lack of personal protective equipment, increased workload, inadequate support, and psychological distress [5]. Furthermore, these studies pointed out the ethical dilemmas they encountered while facing life-threatening situations, such as choosing between personal safety and professional commitment to patient care [6]. Despite these insights, there is a lack of research on how different factors such as experience, tenure, and organisational preparedness might influence nurses' readiness and attitudes towards such extreme healthcare scenarios. We aimed to conduct this study to bridge this knowledge gap and to provide a deeper understanding of nurses’ perspectives in such situations.

Working directly with SARS-CoV-2 infected patients, nurses confronted multiple risks, including the fear of infection, the unpredictability of events, feelings of helplessness, and concerns about performing their professional duties [7]. Existing research has revealed several challenges and dilemmas that nurses faced during the pandemic [8], but no studies have approached this issue from the perspective of nurses in Poland, particularly those in Lublin.

We aimed to explore nurses' views on their work and their capacity to perform professional activities during the COVID-19 pandemic. We selected Lublin for its unique healthcare context, and because it is representative of the broader scenario in Poland. One of our key areas of exploration was the nurses’ sense of security while performing their duties amidst the pandemic.

Our findings should extend our understanding of the challenges nurses face in such crises, support the development of strategies that bolster nurses' professional actions in future DPHEs, and help us prepare our healthcare systems and professionals for future healthcare emergencies.

## METHODS

### Study setting

We recruited participants from several clinical hospitals within the city of Lublin, a significant hub of healthcare provision in the country and one of the largest cities in Eastern Poland, which hosts a variety of healthcare facilities ranging from specialised clinics to general hospitals. Consequently, the healthcare professionals in Lublin have diverse experiences and backgrounds, thus providing a rich source of data for our research. Additionally, the COVID-19 pandemic had a significant impact on this region, making it a viable study setting. Lastly, we selected Lublin due to practical considerations such as accessibility and the researchers' familiarity with the healthcare system in the region, which aided in data collection and interpretation. The selection of these hospitals offering diverse healthcare services, was intended to create a diverse and comprehensive snapshot of the nursing experience during the pandemic. The hospitals included a university children's hospital, a military clinical hospital, a provincial specialist hospital, John of God Hospital, Lublin Oncology Centre, Neuropsychiatric Hospital, Ministry Hospital, and the Institute of Rural Medicine. These facilities provide a representative mix of the different types of healthcare services available in the region. We conducted the study in May and June 2020, during the peak of the COVID-19 pandemic.

### Questionnaire development and validation

We used a self-developed survey questionnaire, constructed following a rigorous and iterative process which began with an exhaustive literature review to better understand the professional and ethical attitudes of nursing staff during the pandemic.

Two researchers searched PubMed, Scopus, and Web of Science for relevant literature published between January 2010 and 2022 using relevant keywords. We excluded conference proceedings, editorials, meeting notes, news items, abstracts, and papers not directly addressing the themes of professional and ethical attitudes in nursing during crises.

Following the literature review, the same two researchers conducted a qualitative thematic analysis of the sourced literature, applying an inductive approach. Each researcher independently identified themes and contrasts which were then discussed collectively until a consensus was reached. We aimed to identify common themes such as “readiness to respond to disaster”, illustrated by willingness to work extra hours, risk exposure, and role flexibility, and “psychological impacts”, reflected in feelings of fear, uncertainty, and helplessness among nurses.

To ensure the content validity and overall comprehensibility, a group of experienced researchers in the field of nursing and healthcare ethics first examined the preliminary version of the survey, evaluating it for relevance of the content selection, appropriateness of the terminology used, exhaustiveness of the topics investigated, the clarity of the survey questions.

After this initial assessment, we piloted the survey with 10 nurses from a clinical hospital in Lublin, asking them to provide their feedback on the relevance, comprehensibility, clarity, and appropriateness of the questionnaire in relation to their practical experiences. Based on the feedback from both the researchers and the nurses, we revised the questionnaire to ensure its overall quality and relevance to the study context.

The resultant questionnaire consisted of nine primary questions designed for completion within five minutes, providing a respondent-friendly experience. Four additional questions quantitatively assessed perceived readiness, focusing on ethical issues related to disaster preparedness. These questions addressed the nurses' perceived risk, personal protection measures, training adequacy, and emotional readiness. These items were formulated as statements to be rated on a five-point Likert scale (range = 1 (strongly disagree) to 5 (strongly agree)), allowing respondents to express their level of agreement or disagreement with each statement, providing nuanced insights into their perspectives.

We selected 10 nurses from a clinical hospital in Lublin to evaluate the preliminary version of the questionnaire. This group was distinct from the main study population and was specifically tasked with assessing the relevance, comprehensiveness, clarity, and appropriateness of the questionnaire to ensure its validity and reliability. We incorporated this group’s feedback into the final version of the questionnaire, but we considered their responses exploratory and did not include them in the final data analysis of the main study.

### Study population and data collection

We conducted this study online due to to the restrictions imposed by the COVID-19 pandemic. After receiving ethical approval from the relevant authorities at the Medical University of Lublin, we sent participation invitations to via email to all nurses working at the Medical University and hospitals in Lublin. We did not consider the racial diversity among the respondents as a variable in this study due to the largely homogenous Polish population in the area. Future studies should involve more diverse populations and consider race as a relevant variable. Although the exact number of email addresses was unavailable due to data protection regulations, we achieved a satisfactory response rate, considering the average response rate for online surveys in the healthcare field. We received 470 responses, and after the exclusion of two responses due to incomplete demographic data, the final sample size for analysis comprised 468 participants.

### Statistical analysis

We conducted all analyses using IBM SPSS Statistics version 23 (IBM, New York, NY, USA). We presented demographic data and overall patterns in the responses using descriptive statistics. We used cross-tabulation to explore relationships between different variables and hypothesis testing to explore any statistical significance. We conducted multiple regression analysis to identify the predictors of perceived readiness to work during the pandemic and χ^2^ tests o explore the association between categorical variables. Before employing these tests, we checked for assumptions of normality, linearity, and homoscedasticity. We considered *P*-values ≤0.05 as statistically significant.

### Ethical considerations

Although the study did not involve a medical experiment, we adhered to strict ethical guidelines. We provided participants with comprehensive information about the study, including its objectives, procedures, potential risks, and benefits, as well as the voluntary nature of participation, the confidentiality of responses, and secure storage of data. We securely stored the data on encrypted servers with restricted access to ensure the confidentiality of participants' information. The participants provided consent upon completing the survey. If participants expressed emotional distress in response to the survey, they were referred to local mental health resources for support.

## RESULTS

The respondents were mainly 35-44 years old (35%), women (88%), and worked primarily in public hospitals (80%). Most nurses had over 20 years of seniority (22%) (**Table 1**). We evaluated their responses in four distinct areas: voluntary service without additional compensation, restricted service due to non-payment, health risks, and freedom of choice restriction.

**Table 1:** Sociodemographic data*

Gender	
Female	414 (88)
Male	54 (12)
Total	468 (100)
**Age**	
Up to 34	134 (29)
35-44 y	163 (35)
45-54 y	122 (26)
55 y and over	49 (10)
Total	468 (100)
**Workplace**	
Public hospital	374 (80)
Research facility	94 (20)
Total	468 (100)
**Length of service**	
0-5 y	92 (20)
6-10 y	71 (15)
11-15 y	100 (21)
16-20 y	101 (22)
More than 20 y	104 (22)
Total	468 (100)

Y – years

*Values presented as mean (%) unless otherwise specified.

### Readiness to be on duty in the event of a disaster even if I am not asked to do so

We surveyed the readiness of respondents to volunteer for on-call duty during a disaster, even in the absence of an explicit request. This measure gauges the depth of their sense of duty and self-initiative during crisis situations.

Most respondents agreed with the prospect of voluntary service, signifying a strong sense of professional commitment. When segmented by gender, female nurses demonstrated a significantly higher willingness to volunteer for extra on-call duties (63% responded “rather yes” or “yes”), showing a robust ethos of service among the participating female nursing staff. We found similar results among the respondents employed in public hospitals; most agreed with the statement (67% responded “rather yes” or “yes”), suggesting a heightened sense of societal responsibility within this group.

Regarding the length of service, we found that the willingness to volunteer for additional on-call duties was most pronounced among participants with the shortest (0-5 years of service, with 71% responding “rather yes” or “yes”) and longest service record (16-20 years of service, with 67% responding “rather yes” or “yes”), implying a commonality of robust professional commitment between these two disparate groups. We observed a different pattern when examining age as a variable. The 45-55-year-old age group was most inclined to disagree with the concept of additional duty (30% responded “no” or “mostly no”); this could indicate greater personal or professional commitments, which could influence the readiness to take on extra duties among nurses in this age category.

We found significant correlations between the declared attitudes of the respondents and their demographic variables, including gender (*P* ≤ 0.002), workplace (*P* ≤ 0.000) age (*P* ≤ 0.01), and length of service (*P* ≤ 0.000) (**Table 2**).

**Table 2:** Readiness to be on duty in the event of a disaster*

							χ^2^, value (df)	*P*-value	Yates’ correction for continuity, value (df)†	*P*-value
**Sex**	**Female**	**Male**	**Total**							
I have no opinion	85 (20)	15 (28)	100 (21)				16.835 (4)	0.002	13.379 (4)	0.009
I disagree	28 (7)	0 (0)	28 (6)							
Rather disagree	42 (10)	13 (24)	55 (12)							
Rather agree	215 (52)	25 (46)	240 (51)							
I agree	44 (11)	1 (2)	45 (10)							
Total	414 (100)	54 (100)	468 (100)							
**Workplace**	**Hospital**	**Research facility**	**Total**							
I have no opinion	72 (19)	28 (30)	100 (21)							
I disagree	23 (6)	5 (5)	28 (6)							
Rather disagree	31 (8)	24 (26)	55 (12)							
Rather agree	205 (55)	35 (37)	240 (51)							
I agree	43 (12)	2 (2)	45 (10)							
Total	374 (100)	94 (100)	468 (100)							
χ^2^							34.379 (4)	0.000		
							31.104 (4)	0.000		
**Age**	**Up to 34 y**	**35-44 y**	**45-54 y**	**55 y and over**	**Total**					
I have no opinion	29 (22)	42 (26)	15 (12)	14 (29)	100 (21)		25.745 (12)	0.01	21.628 (12)	0.04
I disagree	7 (5)	6 (4)	12 (10)	3 (6)	28 (6)					
Rather disagree	10 (7)	15 (9)	25 (20)	5 (10)	55 (12)					
Rather agree	76 (57)	86 (53)	57 (47)	21 (43)	240 (51)					
I agree	12 (9)	14 (8)	13 (11)	6 (12)	34 (10)					
Total	134 (100)	163 (100)	122 (100)	49 (100)	468 (100)					
**Length of service**	**0-5 y**	**6-10 y**	**11-15 y**	**16-20 y**	**More than 20 y**	**Total**				
I have no opinion	14 (15)	22 (31)	27 (27)	7 (7)	30 (29)	100 (21)	44.238 (16)	0.000	37.982 (16)	0.001
I disagree	5 (5)	6 (8.5)	4 (4)	6 (6)	7 (7)	28 (6)				
Rather disagree	8 (9)	8 (11)	8 (8)	20 (20)	11 (11)	55 (12)				
Rather agree	56 (61)	29 (41)	58 (58)	57 (56)	40 (38)	240 (51)				
I agree	9 (10)	6 (8.5)	3 (3)	11 (11)	16 (15)	45 (10)				
Total	92 (100)	71 (100)	10 (100)	101 (100)	104 (100)	468 (100)				

Y – years

*Values presented as n (%) unless otherwise specified.

†We applied Yates’ correction for continuity due to small values for certain parameters.

### Readiness to work overtime in the event of a disaster, even with no pay

We further examined the readiness of nurses to work overtime during a disaster situation, specifically without the promise of extra pay. 

The responses to this question significantly differed from that regarding the nurses’ readiness to be on duty. Most respondents were not in favor of working overtime without pay during a disaster. Male nurses were more likely to reject this proposition (63% responded “rather not” or “no”), indicating a stronger anticipation of financial compensation for their additional work hours. Public hospital employees, who had previously displayed a considerable readiness to volunteer for on-call duties, also commonly disagreed with the idea of working overtime without pay (58% responded “rather not” or “no”), stressing practical considerations of these professionals when asked to sacrifice personal time without a corresponding financial reward.

We found that the youngest age group (<34 years) was more likely to disagree with unpaid overtime work (60% responded “rather not” or “no”), followed closely by 35-44-year-old age group (62% responded “rather not” or “no”). This could infer that younger nurses may be more focused on financial security or have other personal commitments demanding monetary support. Those with 6-10 years of service were the most likely to refuse to work under such conditions (73% responded “rather not” or “no”). This response could be due to a mix of professional expectations and personal circumstances associated with this career stage.

Once again, the correlations between the respondents' stated attitudes and demographic variables were statistically significant (gender: *P* ≤ 0.02; workplace: *P* ≤ 0.000; age: *P* ≤ 0.000; length of service: *P* ≤ 0.000), underlining the impact of these factors on professional attitudes during a crisis situation (**Table 3**).

**Table 3:** Readiness to work overtime in the event of a disaster, even with no pay*

							χ^2^, value (df)	*P*-value	Yates’ correction for continuity, value (df)	*P*-value
**Sex**	**Female**	**Male**	**Total**							
I have no opinion	115 (28)	16 (30)	131 (28)				11.647 (4)	0.02	9.078 (4)	0.05
I disagree	92 (22)	9 (17)	101 (31)							
Rather disagree	133 (32)	25 (46)	158 (34)							
Rather agree	64 (16)	1 (2)	65 (14)							
I agree	10 (2)	3 (5)	13 (3)							
Total	414 (100)	54 (100)	468 (100)							
**Workplace**	**Hospital**	**Research facility**	**Total**							
I have no opinion	108 (29)	23 (24)	131 (28)				27.693 (4)	0.000	23.454 (4)	0.000
I disagree	87 (23)	14 (15)	101 (21)							
Rather disagree	130 (35)	28 (30)	158 (34)							
Rather agree	45 (12)	20 (21)	65 (14)							
I agree	4 (1)	9 (10)	13 (3)							
Total	374 (100)	94 (100)	468 (100)							
**Age**	**Up to 34 y**	**35-44 y**	**45-54 y**	**55 y and over**	**Total**					
I have no opinion	43 (32)	35 (22)	33 (27)	20 (41)	131 (28)					
I disagree	32 (24)	33 (20)	28 (23)	8 (16)	101 (21)					
Rather disagree	48 (36)	69 (42)	30 (25)	11 (23)	158 (34)					
Rather agree	10 (7)	26 (16)	22 (18)	7 (14)	65 (14)					
I agree	1 (1)	0 (0)	9 (7)	3 (6)	13 (3)					
Total	134 (100)	163 (100)	122 (100)	49 (100)	468 (100)		25.745 (12)	0.01	21.628 (12)	0.04
**Length of service**	**0-5 y**	**6-10 y**	**11-15 y**	**16-20 y**	**More than 20 y**	**Total**				
I have no opinion	28 (30)	16 (23)	22 (22)	26 (26)	39 (38)	131 (28)	45.976 (16)	0.000	37.066 (16)	0.002
I disagree	20 (22)	23 (32)	18 (18)	20 (20)	20 (19)	101 (21)				
Rather disagree	34 (37)	29 (41)	40 (40)	36 (35)	19 (18)	158 (34)				
Rather agree	9 (10)	3 (4)	20 (20)	12 (12)	21 (20)	65 (14)				
I agree	1 (1)	0 (0)	0 (0)	7 (7)	5 (5)	13 (3)				
Total	92 (100)	71 (100)	100 (100)	101 (100)	104 (100)	468 (100)				

Y – years

*Values presented as n (%) unless otherwise specified.

†We applied the Yates continuity correction due to small numbers in some values.

### Readiness to be transferred to other departments in the event of disasters

We also assessed the nurses’ willingness to be reassigned to other departments during a crisis. This flexibility is vital since adaptability in roles can greatly influence the overall efficiency and effectiveness of healthcare services in times of disaster.

The respondents displayed a lack of enthusiasm towards the idea of potential transfers. We found a statistically significant correlation only with the variable “length of service” (*P* ≤ 0.000), implying that years of experience and familiarity with a specific work setting could influence nurses' readiness to accept transfers during a crisis. We also found that those with 6-10 years of service frequently disagreed with the proposition of being transferred (55% responded “rather not” or “no”). This response could be due to their settled work rhythm and professional relationships in their current departments, which they might be unwilling to disrupt. Similarly, individuals with 16-20 years of work experience also often disagreed with potential transfers (51% responded “rather not” or “no”). This group, with established professional practices and relationships, might be less inclined to adapt to a new department, even during disaster scenarios.

Our findings indicate that willingness to be transferred to a different department during a crisis is significantly associated with the length of service (**Table 4**).

**Table 4:** Readiness to be transferred to other departments in the event of disasters*

							χ^2^, value (df)	*P*-value	Yates’ correction for continuity, value (df)†	*P*-value
**Length of service**	**0-5 y**	**6-10 y**	**11-15 y**	**16-20 y**	**More than 20 y**	**Total**				
I have no opinion	23 (25)	10 (14)	23 (23)	9 (9)	32 (31)	92 (21)	41.793 (16)	0.000	35.322 (16)	0.003
I disagree	21 (23)	18 (25)	11 (11)	11 (11)	17 (16)	78 (17)				
Rather disagree	24 (26)	21 (30)	38 (38)	41 (40)	18 (17)	142 (30)				
Rather agree	23 (25)	20 (28)	25 (25)	39 (34)	34 (33)	141 (30)				
I agree	1 (1)	2 (3)	3 (3)	1 (3)	3 (3)	10 (2)				
Total	92 (100)	71 (100)	100 (100)	100 (104)	104 (100)	468 (100)				

Y – years

*Values presented as n (%) unless otherwise specified.

†We applied Yates’ correction for continuity due to small values for certain parameters.

### Readiness to take health risks by taking care of people with infectious diseases or exposure to hazardous substances

Finally, we examined the readiness of nursing staff to accept health risks, such as treating patients with infectious diseases or working in environments exposed to hazardous substances. This element of preparedness is crucial during a global pandemic where healthcare professionals are under a heightened risk of exposure to infectious agents.

We found a distinct hesitation towards such risks, especially among less experienced professionals, with a significant correlation only with two demographic variables: length of service (*P* ≤ 0.008) and place of work (*P* ≤ 0.001). More than half of the respondents with less than five years of service were most likely to express their reluctance to take on these risks (57% responded “rather not” or “no”), which could be due to a lack of experience in managing such scenarios or a deficit in confidence to provide care under hazardous conditions. Conversely, public hospital staff often voiced their unwillingness to assume such risks (51% responded “rather not” or “no”), possibly due to perception of a higher exposure risk in public facilities compared to private healthcare settings.

Overall, our results suggest that the fear of health risks and exposure to hazardous substances significantly influences nursing preparedness during a pandemic or disaster (**Table 5**). The readiness to assume such risks is notably associated with the length of service and the type of workplace

**Table 5:** Readiness to take health risks by taking care of people with infectious diseases or exposure to hazardous substances*

							χ^2^, value (df)	*P*-value	Yates’ correction for continuity, value (df)†	*P*-value
**Workplace**	**Hospital**	**Research facility**	**Total**							
I have no opinion	78 (21)	19 (20)	97 (100)				17.649 (4)	0.001	14.590 (4)	0.005
I disagree	69 (19)	9 (10)	78 (100)							
Rather disagree	121 (32)	21 (22)	142 (100)							
Rather agree	101 (27)	40 (43)	141 (100)							
I agree	5 (1)	5 (5)	10 (100)							
Total	374 (100)	94 (100)	468 (100)							
**Length of service**	**0-5 y**	**6-10 y**	**11-15 y**	**16-20 y**	**More than 20 y**	**Total**				
I have no opinion	27 (29)	9 (12)	27 (27)	15 (15)	34 (33)	112 (24)	32.681 (16)	0.008	27.072 (16)	0.040
I disagree	22 (24)	14 (20)	10 (10)	13 (13)	15 14)	74 (16)				
Rather disagree	25 (27)	26 (37)	36 (36)	33 (33)	24 (23)	144 (31)				
Rather agree	17 (19)	20 (28)	25 (25)	39 (38)	28 (27)	129 (27)				
I agree	1 (1)	2 (3)	2 (2)	1 (1)	3 (3)	9 (2)				
Total	92 (100)	71 (100)	100 (100)	101 (100)	104 (100)	468 (100)				

Y – years

*Values presented as n (%) unless otherwise specified.

†We applied Yates’ correction for continuity due to small values for certain parameters.

Overall, a considerable portion of respondents across all four research areas responded with “I have no opinion” to the questionnaire. This suggests a degree of uncertainty or reluctance to assert their attitudes towards potential challenges during a disaster or pandemic.

In the first research area, focusing on readiness to be on-call during a disaster even without being directly requested, most respondents expressed willingness (responding “rather yes” or “yes”; n = 285 (61%)), reflecting a general inclination among the nursing staff to step up during crisis times. However, the scenario changed when asked about working overtime without pay during a disaster, with more than half of the respondents disagreeing (“no” or “mostly no”; n = 259 (55%)). This highlights the need for compensation and acknowledgement for extra work hours, particularly during challenging situations such as a pandemic.

When it comes to assuming health risks by treating infectious patients or working in hazardous environments, less than half of the respondents were agreeable (“no” or “mostly no”; n = 218 (47%)). A similar proportion disagreed with the prospect of being transferred to other departments during a disaster (“no” or “mostly no”; n = 220 (47%)). These results reveal a resistance to changes in the usual work environment and practices and the additional health risks such changes might pose.

We observed a highly statistically significant relationship was found between respondents’ declared attitudes (*P* ≤ 0.000) across all four research areas. While we observed the response “I don't have an opinion” was frequent among the oldest and most experienced respondents, we did not find a statistically significant relationship to conclude that they might be more hesitant to express definitive opinions on these matters.

**Figure 1:**
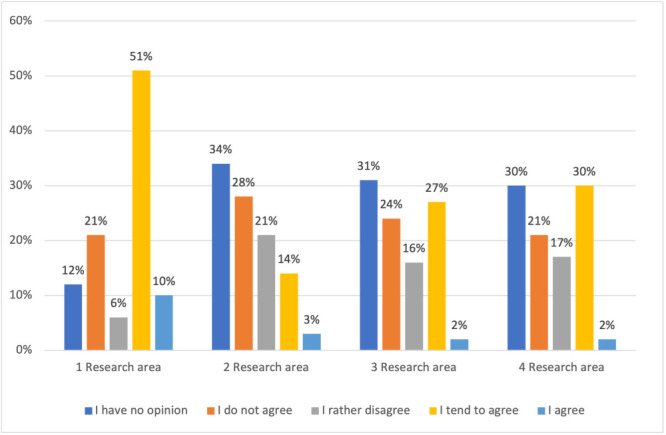
Frequency distribution of respondents' answers by research area.

This comprehensive view of the respondents' attitudes and perceptions offers valuable insights for decision-makers in healthcare planning and policy, particularly in the context of disaster management and pandemic response (**Figure 1**).

## DISCUSSION

As per the World Health Organization's (WHO) the State of the world's nursing report, nurses make up more than half of all health professionals worldwide, tirelessly providing care to patients regardless of their health and social circumstances [9].

However, the unique working conditions during the COVID-19 pandemic introduced new demands and challenges for this group [10]. IN the early phase of the pandemic, many nurses were under immense strain due to a lack of personal protective equipment, overwhelming numbers of patients, staff shortages, overtime, and unprepared healthcare systems. The nurses also had to tackle complex ethical dilemmas and moral conflicts while working under constant stress and caring for patients suspected or confirmed to be infected with SARS-CoV-2 [11,12].

According to the American Nurses Association's Code of Ethics, nurses' primary duty is to provide care to an individual, a family, or a community. However, the same Code also mandates them to take care of their health and safety [2,13,14], which became challenging during the pandemic.

In a disaster scenario like the COVID-19 pandemic, providing nursing care can be extraordinarily stressful and demanding [13-15]. Chronic stress at work can induce anxiety, decrease job satisfaction, and ultimately lead to job burnout. According to Grzelak et al., [15] 6.2% of surveyed nurses reported recurrent thoughts of leaving the profession. A separate report found 6.3% of nurses considering leaving the profession and a 3.8% considering migration [16].

In our study, 61% of respondents stated that they were prepared to serve during a disaster, even without an explicit request. The respondents most willing to do so were aged up to 34 years (68%). Respondents with the least amount of service (0-5 years; 71%) were more willing to be on-call during a disaster than those with longer service lengths. This willingness was most prominent among women (63%), which aligns with the female-majority demographic of the nursing profession in Poland.

The COVID-19 pandemic revealed a global systemic healthcare weakness: a shortage of medical staff [17,18], particularly nurses, which was also observed in Poland [19]. Overworked nurses were required to work under high stress and personal risk conditions [20,21]. Evidence shows that heavier workloads correlate with higher stress levels and greater unwillingness to work additional hours, especially if unpaid [22]. Consequently, the greater the challenges, the fewer the staff available. This overloading of tasks can lead to constant exhaustion and depression, which was evident among Polish nurses during the pandemic [20].

Nurses highly committed to their profession who do not receive adequate remuneration are at risk of professional burnout, and mental and somatic health disorders. In our survey, over half of the respondents (55%) were unwilling to work overtime without pay in the event of a disaster. This reluctance was more common in men (63%) and employees of public hospitals (58%), which form the backbone of healthcare during disasters. Reports suggest that nurses are more susceptible to COVID-19 infection than other healthcare workers and the general population [22-25]. Shortages of personal protective equipment or testing kits, particularly during the early stages of the pandemic, exacerbated their fears. The lack of adequate protective measures not only risked the health of the nurses but also exposed patients to the risk of infection, raising social justice issues and ethical dilemmas [26,27].

Jia et al. [28] emphasised the disparity in exposure to infectious environments between nurses and doctors, with nurses' exposure being considerably higher. In our survey, 47% of respondents were not willing to take health risks when caring for infectious patients or when exposed to hazardous substances. This sentiment was most common among respondents with 6-10 years of service (57%).

During the COVID-19 pandemic, nurses felt the weight of additional roles and responsibilities. They were often expected to adapt to new duties, with doctors expecting nurses to shoulder some of their responsibilities [29]. This expectation, alongside disparities in professional training, knowledge, and experience, caused moral conflicts and constraints [30,31].

Assigning intensive care duties related to COVID-19 to nurses without specialized training is unadvisable due to the increased risk of errors. However, enhancing a nurse's knowledge could improve their confidence and willingness to work with COVID-19 patients [32]. It's important to note that nurses should never be forced to perform tasks they are not comfortable with [33,34]. The WHO COVID-19 guidelines even allow healthcare employees to withdraw from work situations if they have reasonable justification [35].

In our survey, 47% of respondents were not ready to be transferred to other units during a disaster, particularly among respondents with 6-10 years of service (55%). Any change, such as a transfer to another unit during a disaster, should align with the nurse's assessment of acceptable risk and their training level.

Our findings should be interpreted with regards to several limitations. First, the study was geographically confined to nurses in Lublin city, which is a major limitation. Consequently, we cannot generalise our findings to the perspectives and experiences of nurses from other regions and countries, each with their own socio-economic contexts and healthcare systems.

We conducted this study during the COVID-19 pandemic, which imposed substantial challenges on our data collection process. We conducted the survey online due to safety precautions that limited our physical access to staff. Although this method offered a safe and efficient avenue for data collection, it may have influenced the response rates and our overall results. Notably, some nurses less adept or comfortable with online tools might have been less inclined to respond, potentially skewing our sample towards a more tech-savvy population. Moreover, we dd not consider personal factors that could have influenced the participants’ responses, such as existing or chronic health conditions, pregnancy status, and pressures from family or spouses. All these factors could significantly impact an individual's willingness to shoulder certain responsibilities and risks in a disaster situation.

Moreover, our questionnaire did not incorporate open-ended questions. This lack of qualitative data collection restricted respondents from articulating their thoughts and worries in their own words or bringing up issues they felt were relevant but were not covered in the questionnaire. We also faced a significant limitation in our inability to accurately determine the participation rate and evaluate the representativeness of our sample. Due to logistical hurdles, we could not determine the total number of eligible nurses, making it unclear what proportion of the target population our sample represents.

Despite these limitations, this study initiates a critical dialogue on the attitudes and readiness of nurses during disaster situations and underscores the need for further exploration in this field, contributing to the future development of more comprehensive and standardised research tools.

## CONCLUSIONS

We found that a significant proportion of nurses are prepared to make substantial personal sacrifices in times of disaster, while generally being ready to accept the high levels of personal risk linked with working during a pandemic, such as COVID-19. Factors such as excessive workload, fear of infection, and feelings of helplessness can undermine their willingness to work overtime, particularly if unpaid. This suggests that healthcare administrators should prioritise establishing suitable and safe working conditions., including the provision of adequate personal protective equipment, which not only assures the safety of nurses, but also of the patients are caring for. Extending comprehensive insurance coverage for adverse events and third-party liability against the transmission of infectious diseases, including SARS-CoV-2, presents another vital measure that could enhance nurses' readiness.

Additionally, healthcare managers must ensure that nurses have access to robust systemic psychological support to help them navigate circumstances where their health and lives are under threat. This support should be conveniently available and tailored to address the unique stresses and dilemmas that nurses confront during a pandemic or other healthcare crises. Aside from these logistical considerations, our study also underscores the ethical dilemmas nurses may grapple with in their roles. These issues should inform and guide healthcare professionals in their decision-making process, highlighting the need for ethical guidelines custom-fit for crisis situations.

These measures must undergo regular evaluation through similar studies to guarantee the ongoing adaptability and resilience of our healthcare system. The insights derived from such research can equip us to better prepare for future crises, ensuring our nurses and other healthcare professionals are supported, protected, and empowered to deliver optimal care, even under the most challenging circumstances.
